# Establishment of non-small-cell lung cancer risk prediction model based on prognosis-associated ADME genes

**DOI:** 10.1042/BSR20211433

**Published:** 2021-10-14

**Authors:** Ke Han, Jukun Wang, Kun Qian, Teng Zhao, Yi Zhang

**Affiliations:** 1 Department of Thoracic Surgery, Xuanwu Hospital of Capital Medical University, No. 45 Changchun Street, Xicheng District, Beijing 100053, China; 2 Department of General Surgery, Xuanwu Hospital of Capital Medical University, No. 45 Changchun Street, Xicheng District, Beijing 100053, China

**Keywords:** ADME gene, Non–small-cell lung cancer, prognostic factor, risk model

## Abstract

**Purpose:** ADME genes are those involved in the absorption, distribution, metabolism, and excretion (ADME) of drugs. In the present study, a non-small-cell lung cancer (NSCLC) risk prediction model was established using prognosis-associated ADME genes, and the predictive performance of this model was evaluated and verified. In addition, multifaceted difference analysis was performed on groups with high and low risk scores.

**Methods:** An NSCLC sample transcriptome and clinical data were obtained from public databases. The prognosis-associated ADME genes were obtained by univariate Cox and lasso regression analyses to build a risk model. Tumor samples were divided into high-risk and low-risk score groups according to the risk score. Gene Ontology and Kyoto Encyclopedia of Genes and Genomes enrichment analyses of the differentially expressed genes and the differences in the immune infiltration, mutation, and medication reactions in the two groups were studied in detail.

**Results:** A risk prediction model was established with seven prognosis-associated ADME genes. Its good predictive ability was confirmed by studies of the model's effectiveness. Univariate and multivariate Cox regression analyses showed that the model’s risk score was an independent prognostic factor for patients with NSCLC. The study also showed that the risk score closely correlated with immune infiltration, mutations, and medication reactions.

**Conclusion:** The risk prediction model established with seven ADME genes in the present study can predict the prognosis of patients with NSCLC. In addition, significant differences in immune infiltration, mutations, and therapeutic efficacy exist between the high- and low-risk score groups.

## Introduction

Currently, lung cancer remains the leading cause of cancer-related death worldwide, and it represents both the first and second highest incidences and mortality rates among men and women [1]. Lung cancer is divided into small-cell lung cancer (approximately 15%) and non-small-cell lung cancer (NSCLC, approximately 85%). NSCLC includes lung adenocarcinoma (LUAD), lung squamous cell carcinoma (LUSC), and lung large cell carcinoma [2]. With the development of epidermal growth factor tyrosine kinase inhibitors, immunoassay checkpoint inhibitors, and other drugs, great progress has been made in the treatment of lung cancer, especially LUAD. However, these treatments offer limited benefits, and drug resistance, followed by disease progression, develops 10–12 months after medication begins [3]. Therefore, a deep understanding of the relevant regulatory mechanisms of drug metabolism in tumors is essential.

ADME genes are a group of genes involved in the absorption, distribution, metabolism, and excretion (ADME) of drugs. They include phase I (functionalization) and phase II (conjugation) drug-metabolizing enzymes, transporters, and modifiers, and they are involved in drug metabolism and drug clearance by the liver. Currently, the PharmaADME Consortium has defined 298 ADME genes, including 32 core ADME genes and 266 extended ADME genes [4,5]. ADME genes are involved in the regulation of metabolism, transportation, and removal of endogenous and exogenous substances-processes that may be associated with tumorigenesis and tumor development. A previous study has shown that ADME genes are significantly differently expressed between lung cancer tissues and normal lung tissues and are closely correlated with the prognosis of patients with lung cancer [6,7]; furthermore, ADME genes have the potential to be prognostic biomarkers and therapeutic targets for cancer [8]. It is not difficult to speculate that ADME genes may influence the curative effect of lung cancer treatments as well as the tumor microenvironment composition itself. No study, to our knowledge, has examined patients with lung cancer with respect to an NSCLC risk prediction model established on the basis of ADME genes.

In the present study, the mRNA expression data and relevant clinical information of patients with NSCLC were retrieved from public databases. Prognosis-associated ADME genes were used to establish a risk prediction model, the predictive ability of which was verified by a Gene Expression Omnibus (GEO) cohort. The study also analyzed the functional enrichment of differentially expressed genes (DEGs) and the differences in immune infiltration, mutation, and medication reactions between high- and low-risk score groups.

## Materials and methods

### Data collection

The RNA sequencing (RNA-seq) of NSCLC (LUAD + LUSC) tumor samples and the corresponding clinical data were obtained from The Cancer Genome Atlas (TCGA) database (https://portal.gdc.cancer.gov/) as a training set. The survival information for patients who provided NSCLC samples was obtained from the GSE30219 chip sequence in the GEO database (https://www.ncbi.nlm.nih.gov/geo/). ADME genes (*n*=298) were obtained from previous literature [8].

### DEGs of prognosis-associated ADME genes obtained from the training set

The DEGs in NSCLC tumor tissues and normal tissues were screened with the “limma” package in R software (Ver. 3.6.1) (https://www.r-project.org/). The screening criteria for differential mRNA were a |log2FoldChange| > 1 and an adjusted *P*<0.01. A volcano plot was drawn with the “ggplot2” R package. The intersection between DEGs and ADME genes was taken with the “Venn” package. Candidate ADME genes were obtained, and a Venn diagram was drawn for a visual display. The ADME genes significantly associated with the prognosis of NSCLC were screened with univariate Cox regression analysis.

### Network and correlation analysis of prognosis-associated ADME genes

The protein–protein interaction (PPI) network of prognosis-associated ADME genes was obtained from the String database (http://string-db.org/). The correlation coefficient between network nodes was calculated, and the co-expression of prognosis-associated ADME genes was analyzed.

### Establishment and efficiency verification of a risk prediction model based on prognosis-associated ADME genes

The lasso regression analysis of the R package “glmnet” was used for dimensionality reduction and calculation of the coefficient of prognosis-associated ADME genes. The risk score of each sample was obtained using a specific formula, as follows:

$$\text{Risk}\;\text{score}=\beta_{1}\times {\text{Exp}}_{1}+\beta_{2}\times {\text{Exp}}_{2}+\beta_{\text{i}}\times {\text{Exp}}_{\text{i}}$$

In which β is the regression coefficient and Exp is the expression level of ADME genes. Patients with NSCLC were divided into a high-risk group and a low-risk group according to the median risk score. Then, “survival” and “survivalROC” packages were used to analyze the survivorship and receiver operating characteristic (ROC) curves, and the “prcomp” package was used for principal component analysis (PCA) and T-distributed stochastic neighbor embedding (t-SNE) analysis to determine the distribution of genes in different groups and to clarify the accuracy of the risk prediction model. In addition, the NSCLC samples in the GEO database were used as a validation set to verify the predictive performance of the model.

### Functional enrichment analysis for DEGs in high- and low-risk score groups

The samples included in TCGA and GEO databases were divided into high- and low-risk score groups on the basis of the median risk score. The DEGs in the two groups were calculated with the “limma” package. The screening criteria were a |log2FoldChange| > 1 and a false discovery rate < 0.01. The “Cluster Profiler” package was used for the Gene Ontology (GO) enrichment analysis of DEGs to determine their biological functions in the enrichment of biological processes, cellular component, and molecular function. Kyoto Encyclopedia of Genes and Genomes (KEGG) functional enrichment analysis was performed on DEGs.

### Analysis of immune infiltration differences between high- and low-risk score groups and correlation between risk score and immune cells

The immune score of the immune cell type in the samples from TCGA was estimated using the CIBERSORT method. The box diagram of immune infiltration was drawn with the R package “gsva” by means of a single-sample gene set enrichment analysis (ssGSEA). The infiltration level of 23 types of immune cells was determined. Then, the correlation between the risk score and the immune infiltration score of different cell types was analyzed.

### Analysis of mutation differences and evaluation of medication reactions in high- and low-risk score groups

The single-nucleotide polymorphism (SNP) file of the training set data was analyzed, and the mutation difference in candidate ADME gene-associated DEGs (obtained from the intersection between DEGs and ADME genes) between the high- and the low-risk score groups was determined. Tumor immune dysfunction and exclusion (TIDE) analysis (http://tide.dfci.harvard.edu/login/) was conducted on the high- and the low-risk score groups to predict the difference in the effect of immunotherapy on samples between the two groups. The Fisher exact test was performed according to response and nonresponse to treatment in the high- and the low-risk score groups to evaluate the difference in medication reactions between the two groups.

### Statistical analysis

All statistical analyses were performed with R software (Ver. 3.6.1). The DEGs in tumor tissues and normal tissues were compared using the Wilcoxon test. The survivorship curve was generated by the Kaplan–Meier method; the significance of difference was determined by log-rank test. The ROC curve and the area under the ROC curve (AUC) were used to evaluate the accuracy of the risk prediction model. Univariate and multivariate Cox regression analyses determined the independent prognostic factor for patients with NSCLC. *P*<0.05 was considered statistically significant.

## Results

**Figure 1:**
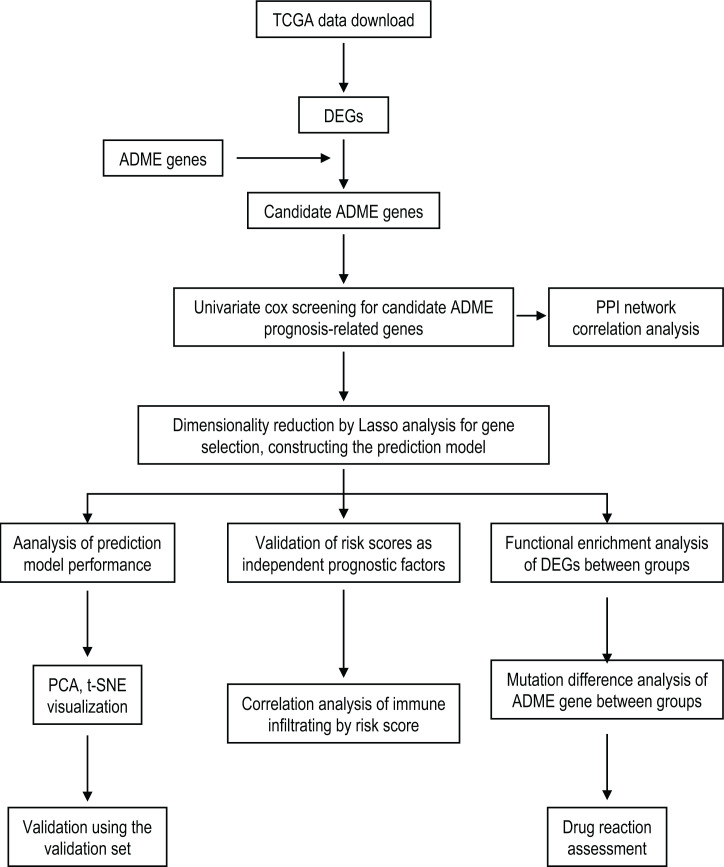
Flow chart of data analysis

As shown in the flowchart (Figure 1), the training set comprised the RNA-seq and clinical data of the 1019 tumor samples and 110 normal samples from TCGA. Supplementary Material S1 lists the detailed clinical features of these samples. The validation set data comprised the survival information of the 293 NSCLC samples from the GSE30219 chip sequence of the GEO database, and the survival information of 293 NSCLC patients was presented in Supplementary Material S2.

### Screening of differentially expressed ADME genes

**Figure 2:**
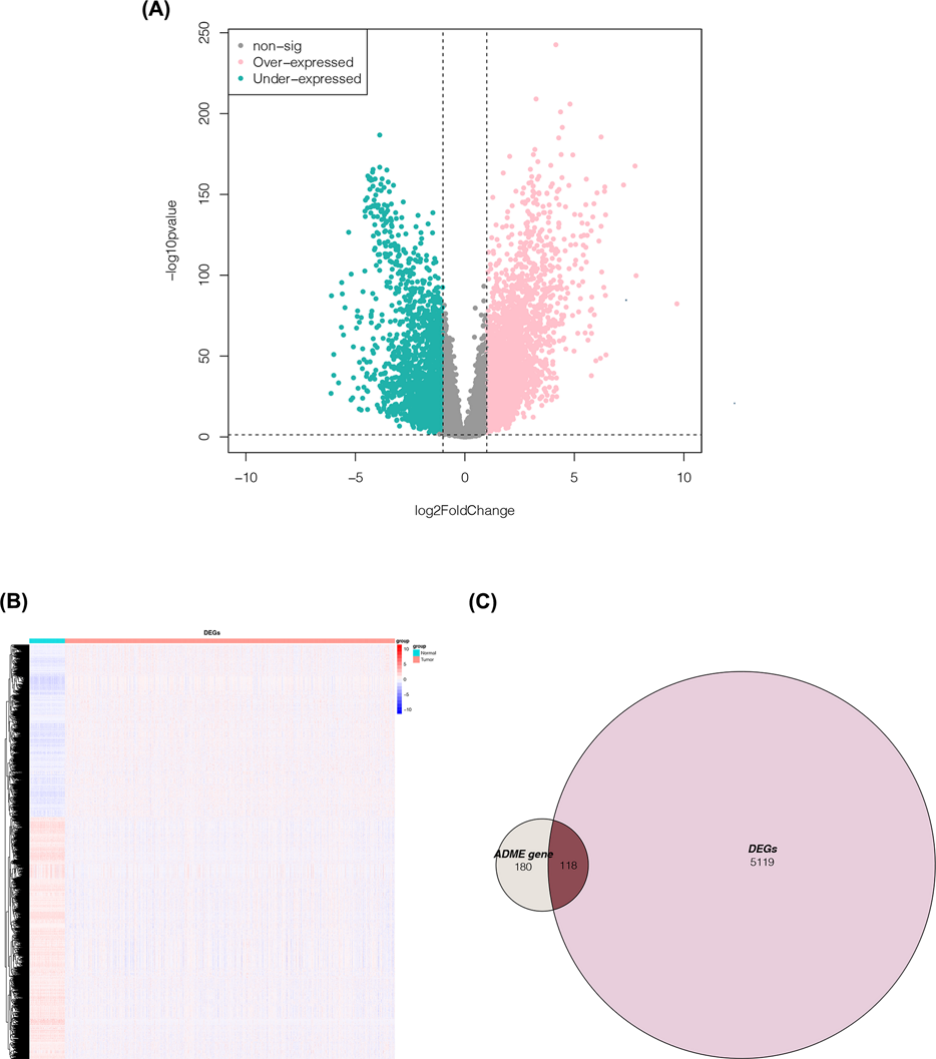
Acquisition of candidate ADME genes (**A**) Volcano plot of DEGs between NSCLC tumor and normal tissues in training set. The *X*-axis is log2(FC); the *Y*-axis is −log10(*p*). (**B**) Characteristic heat maps of DEGs. Rows are genes; columns are samples. (**C**) Venn diagram obtained by taking the intersection between DEGs and ADME genes.

A difference analysis was performed on the RNA-seq data of the tumor samples and normal tissue samples from TCGA. The 5237 DEGs obtained are shown in a volcano plot (Figure 2A), In addition, a list of 5237 DEGs was presented in Supplementary Material S3. The characteristic heat maps of these DEGs show the expression differences between tumor tissues and normal tissues (Figure 2B). The intersection between DEGs and 298 ADME genes was also taken. One hundred eighteen candidate ADME genes were obtained for subsequent analysis. A Venn diagram was drawn for a visual display (Figure 2C).

### Determination of ADME genes associated with the prognosis of NSCLC by univariate Cox regression analysis

**Figure 3:**
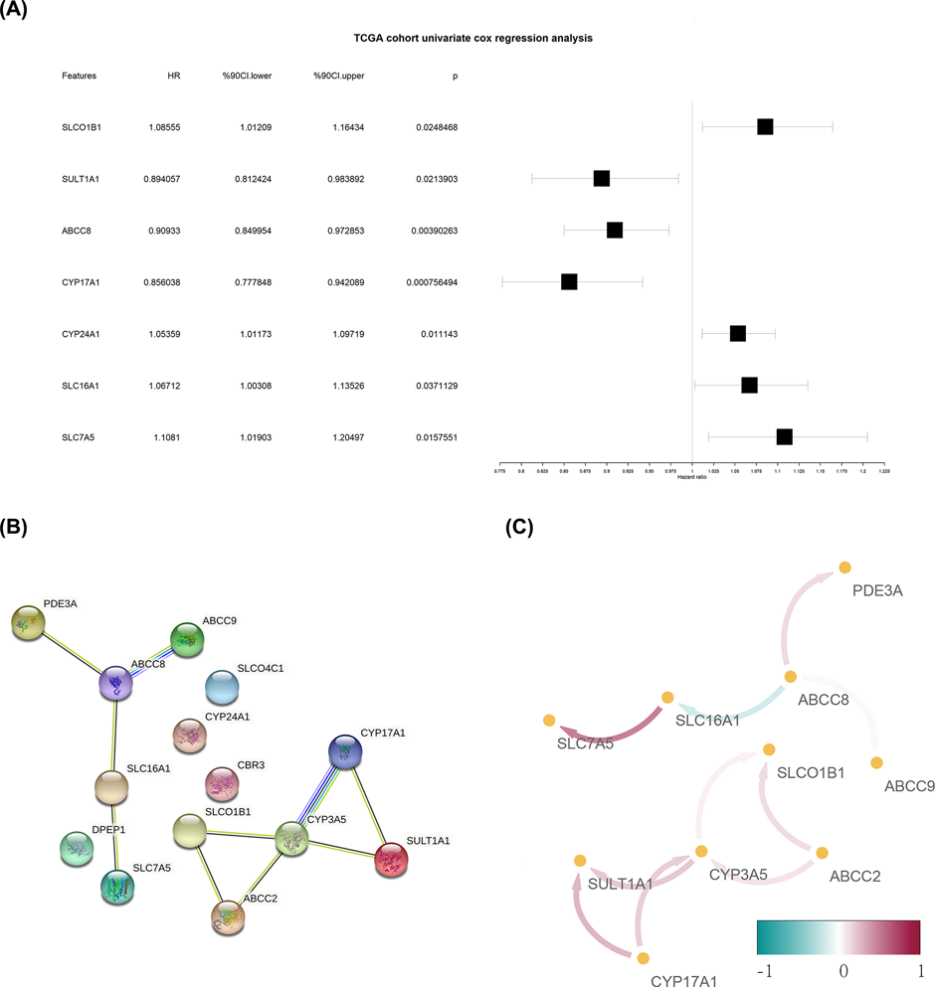
Acquisition of prognosis-associated ADME gene (**A**) Forest plot shows candidate ADME genes. Prognosis-associated ADME genes were obtained by univariate Cox regression analysis. (**B**) PPI network diagram of prognosis-associated ADME genes. (**C**) Co-expression diagram for prognosis-associated ADME genes. The color of the side represents the co-expression value between two ADME genes. The darker the color, the greater the correlation. Red represents a positive correlation; green represents a negative correlation.

The correlation between 118 candidate ADME genes and the overall survival (OS) of patients with NSCLC was studied by univariate Cox regression analysis. Fourteen genes significantly associated with the prognosis of NSCLC (*PDE3A, ABCC8, ABCC9, SLCO4C1, CYP24A1, SLC16A1, DPEP1, SLC7A5, CBR3, SLCO1B1, ABCC2, CYP3A5, CYP17A1*, and *SULT1A1*) were obtained (Figure 3A).

The PPI network of the 14 prognosis-associated ADME genes was established in the String database. A close interaction between the proteins corresponding to such genes was revealed (Figure 3B). The correlation analysis showed that prognosis-associated ADME genes were co-expressed. In particular, *SLC7A5* was positively correlated with *SLC16A1* (Figure 3C).

### Establishment of an NSCLC risk prediction model based on prognosis-associated ADME genes

**Figure 4:**
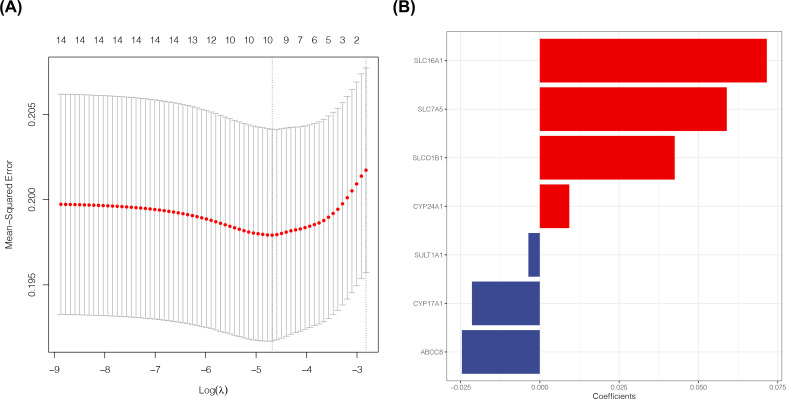
Acquisition of prognosis-associated ADME genes for establishing an NSCLC risk prediction model (**A**) Lasso regression analysis was used for dimensionality reduction for the 14 prognosis-associated ADME genes. The *X*-axis shows λ; the *Y*-axis shows error. (**B**) Coefficient diagram for seven prognosis-associated ADME genes obtained from lasso regression analysis. Red represents a positive value; and blue represents a negative value.

**Figure 5:**
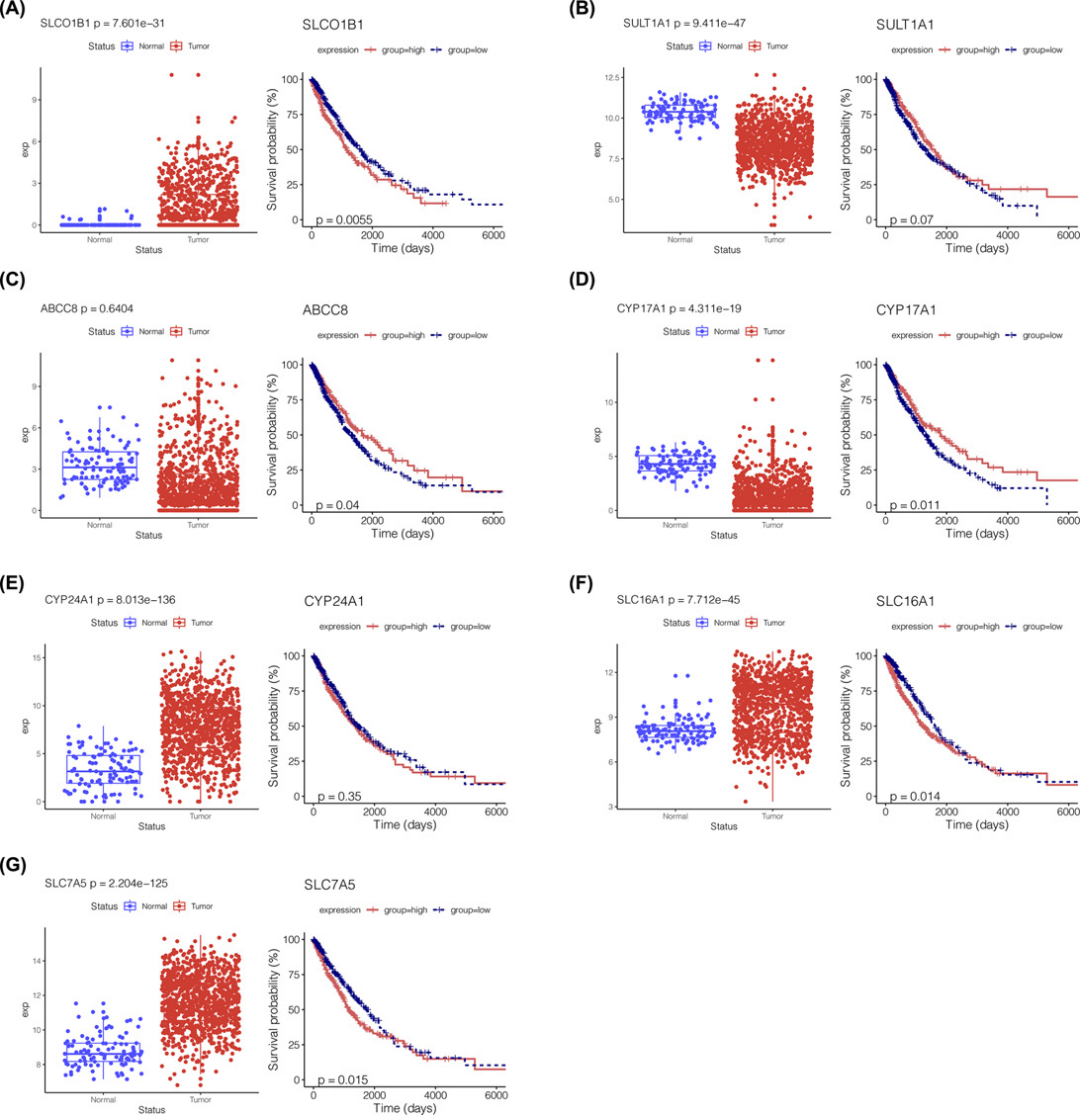
Survival analysis of prognosis-associated ADME genes for establishing an NSCLC risk prediction model (**A–G**) The box plot shows the difference in the expression of the seven prognosis-associated ADME genes between tumor and normal tissues. The Kaplan–Meier survivorship curve shows the correlation between the expression of the seven prognosis-associated ADME genes and the survival of patients with NSCLC.

Lasso regression analysis was used for dimensionality reduction on the 14 prognosis-associated ADME genes and to calculate the prognosis coefficient, thus obtaining the risk score of samples (Figure 4A). Seven ADME genes (*SLC16A1, SLC7A5, SLCO1B1, CYP24A1, SULT1A1, CYP17A1*, and *ABCC8*) were retained to establish the risk prediction model (Figure 4B). The difference in the expression of the five ADME genes (*SLC16A1, SLC7A5, SLCO1B1, CYP17A1*, and *ABCC8*) between tumor and normal tissues was significant and was closely correlated with the survival of patients with NSCLC (Figure 5).

### Efficiency evaluation on risk prediction model

**Figure 6:**
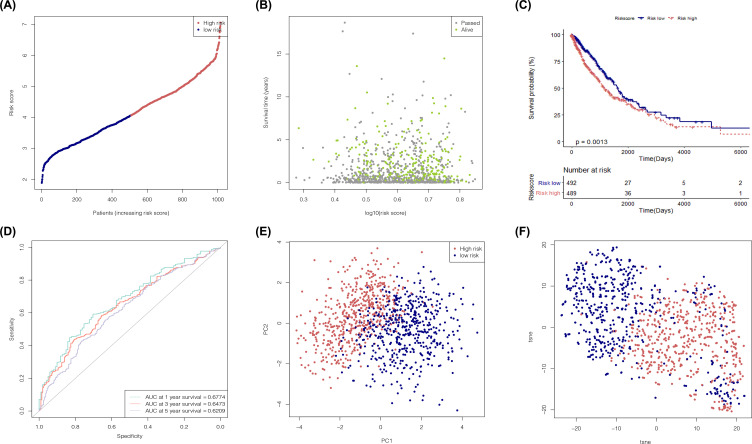
Efficiency analysis on risk prediction model (**A**) Distribution diagram of risk score of NSCLC tumor samples in training set. (**B**) Risk map of tumor samples in training set. The *X*-axis represents the risk score; the *Y*-axis represents the survival time. (**C**) Survivorship curve of high- and low-risk score groups for tumor samples in the training set. (**D**) Prognosis prediction ROC curve 1, 3, and 5 years after treatment of patients with NSCLC in the training set. (**E** and **F**) PCA diagram and t-SNE diagram show the risk score groups of the tumor samples in the training set.

The samples in the training set were divided into a high-risk group (*n*=506) and a low-risk group (*n*=513) (Figure 6A), and the median risk score of the risk prediction model was the threshold. Both the risk map and the Kaplan–Meier curve chart showed that the survival time of patients in the low-risk group was significantly longer than that of patients in the high-risk group (Figure 6B,C). The ROC curves (AUC_1_ = 0.677, AUC_3_ = 0.65, and AUC_5_ = 0.621) (Figure 6D) showed that this risk prediction model had good predictive ability for the survival state of patients with NSCLC in years 1, 3, and 5. The PCA (Figure 6E) and t-SNE (Figure 6F) analyses showed significant differences in the spatial position between the high-risk group and the low-risk group, indicating that this model could make a good distinction between those at high risk of NSCLC and those at low risk.

### Efficiency evaluation of the risk prediction model by the validation set

**Figure 7:**
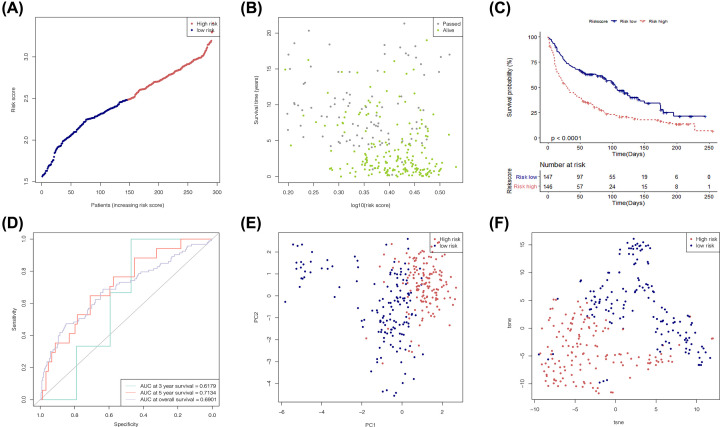
Efficiency analysis of risk prediction model with data in the training set (**A**) Distribution diagram of risk score of NSCLC tumor samples in the validation set (GSE30219). (**B**) Risk map of tumor samples in the validation set. The *X*-axis represents the risk score; the *Y*-axis represents the survival time. (**C**) Survivorship curve of high- and the low-risk score groups for tumor samples in the validation set. (**D**) Prognosis prediction ROC curve of patients with NSCLC in the validation set. (**E** and** F**) PCA diagram and t-SNE diagram show the risk score groups of the tumor samples in the validation set.

According to the median risk score obtained from the training set, the samples in the validation set were divided into a high-risk group (*n*=150) and a low-risk group (*n*=143) (Figure 7A). Both the risk map and the Kaplan–Meier curve chart showed that the survival time of patients in the high-risk group was significantly shorter than that of patients in the low-risk group (Figure 7B,C). The ROC curves (AUC_3_ = 0.62, AUC_5_ = 0.71, and AUC_os_ = 0.69) (Figure 7D) showed that this risk prediction model had good predictive ability for the survival state of patients with NSCLC. The PCA (Figure 7E) and t-SNE (Figure 7F) analyses showed significant differences in the spatial position between the low-risk group and the high-risk group, indicating that this model could make a good distinction between the high- and low-risk score groups.

### Risk score as an independent prognostic factor for patients with NSCLC

**Figure 8:**
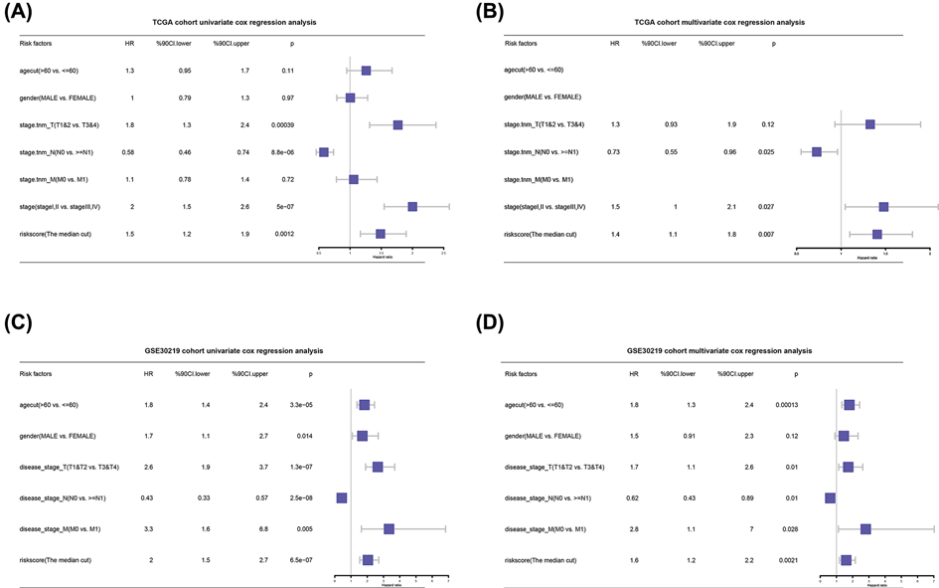
Risk score as an independent prognostic factor for patients with NSCLC (**A** and** C**) Forest plot obtained from univariate Cox regression analysis in the training set/validation set shows the clinical features of NSCLC and the relation between risk score and prognosis. (**B** and **D**) Forest plot obtained from multivariate Cox regression analysis in the training set/validation set shows the clinical features of NSCLC and the relation between risk score and prognosis.

The forest plot drawn by the univariate Cox regression analysis showed that the risk score is an independent prognostic factor for patients with NSCLC. In the training set (Figure 8A), the hazard ratio (HR) of the risk score was 1.5 (95% CI: 1.2–1.9, *P*=0.001). Other clinical features, such as neoplasm staging, lymph node staging, and tumor stage, also were independent prognostic factors of patients with NSCLC. In the validation set (Figure 8C), the HR of the risk score was 2 (95% CI: 1.5–2.7, *P*=6.5e-07). Age, gender, TNM staging, and tumor stage were independent prognostic factors for patients with NSCLC. When multivariate Cox regression analysis was performed on the training set (Figure 8B), the HR of the risk score was 1.6 (95% CI: 1.2–2.1, *P*=0.002). Among other clinical features, lymph node staging was an independent prognostic factor for patients with NSCLC. In the validation set (Figure 8D), the HR of the risk score was 1.6 (95% CI: 1.2–2.2, *P*=0.002). Among other clinical features, age, TNM staging, and tumor stage were independent prognostic factors for patients with NSCLC.

### Functional enrichment analysis for DEGs in high- and low-risk score groups

**Figure 9:**
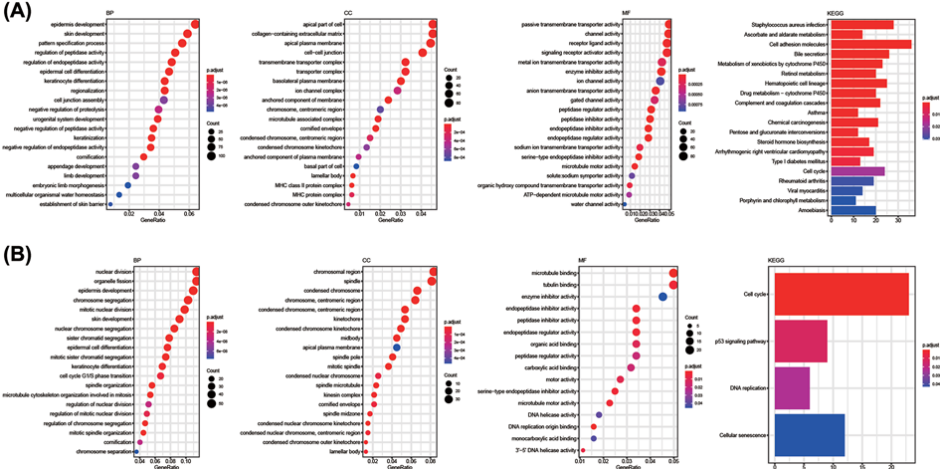
Functional enrichment analysis for DEGs in high- and low-risk score groups (**A** and** B**) GO (including BP, CC, and MF analyses) and KEGG function enrichment analysis of DEGs in the high- and the low-risk score groups in the training set/validation set. The redder the color, the greater the enrichment *P* value.

The relation between the biological function of the DEGs in the high- and low-risk score groups and the risk score of ADME genes was explored by GO and KEGG enrichment analyses. Overall, 2171 and 531 DEGs were identified in the training set and the validation set, respectively. GO enrichment analysis showed that the DEGs in the high- and the low-risk score groups were enriched in several metabolism-related molecular pathways, such as enzyme inhibitor activity, peptidase regulator activity, peptidase inhibitor activity, endopeptidase inhibitor activity, endopeptidase regulator activity, and serine-type endopeptidase inhibitor activity (Figure 9). KEGG enrichment analysis showed that DEGs were also enriched in metabolism-related pathways, such as drug metabolism by cytochrome P450 and metabolism of xenobiotics by cytochrome P450. DEGs were also enriched in tumor-related pathways, such as the cell cycle, the p53 signaling pathway, DNA replication, and chemical carcinogenesis (Figure 9).

### Analysis of differences in immune cell infiltration between high- and low-risk score groups and correlation between risk score and immune cells

**Figure 10:**
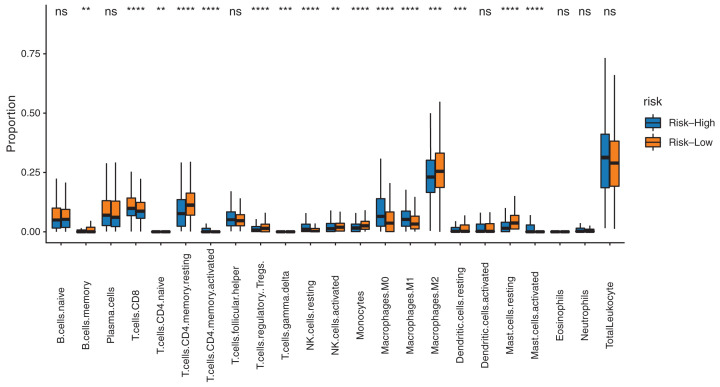
Analysis of differences in immune cell infiltration between high- and low-risk score groups Distribution of immune cells in samples in the high- and the low-risk score groups. The *X*-axis shows the type of immune cells; the *Y*-axis shows the percentage of cells.

**Figure 11:**
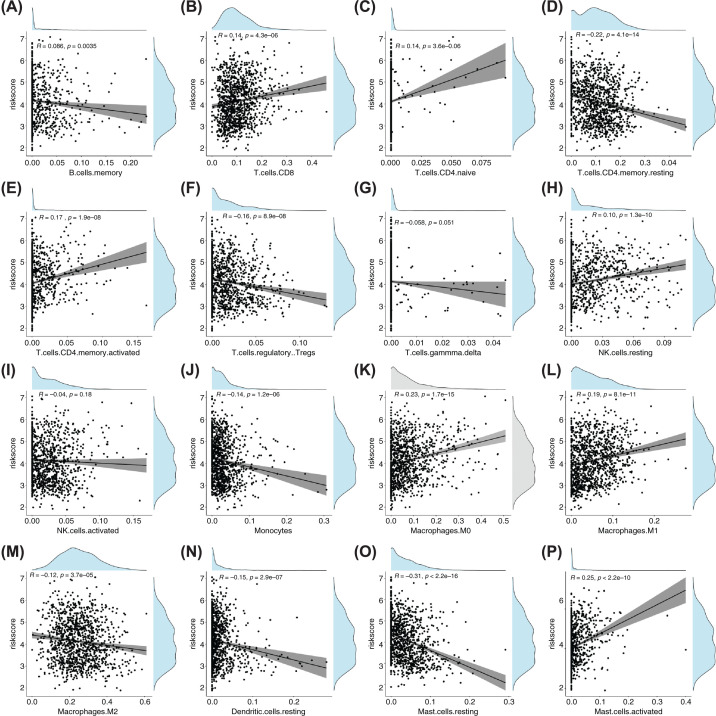
Correlation between risk score and immune cells (**A–P**) Correlation between 16 types of immune cell infiltration and risk scores. The *X*-axis shows the proportion of cells; the *Y*-axis shows the risk score.

In the training set, the box plot drawn from the immune infiltration score of samples in the high- and the low-risk score groups (Figure 10) showed a significant difference in multiple immune infiltrations. In the low-risk score group, the levels of the following immune infiltrations were significantly elevated: memory B cells, CD4 memory resting T cells, regulatory T cells, gamma delta T cells, activated natural killer (NK) cells, monocytes, resting dendritic cells, and resting mast cells. In the high-risk score group, the levels of the following immune infiltrations were elevated: CD8 T cells, CD4-naïve T cells, activated CD4 memory T cells, resting NK cells, M0 macrophages, M1, macrophages, M2 macrophages, and activated mast cells. The results of the correlation test on the risk score and the immune infiltration score of different immune cell types showed that the risk score was positively correlated with the immune infiltration of CD8 T cells, CD4-naïve T cells, activated CD4 memory T cells, resting NK cells, M0 macrophages, M1 macrophages, and activated mast cells, whereas the risk score was negatively correlated with the immune infiltration of memory B cells, resting CD4 memory T cells, regulatory T cells, gamma delta T cells, activated NK cells, monocytes, resting dendritic cells, resting mast cells, and M2 macrophages (Figure 11).

### Analysis of mutation differences and evaluation of medication reactions in the high- and low-risk score groups

**Figure 12:**
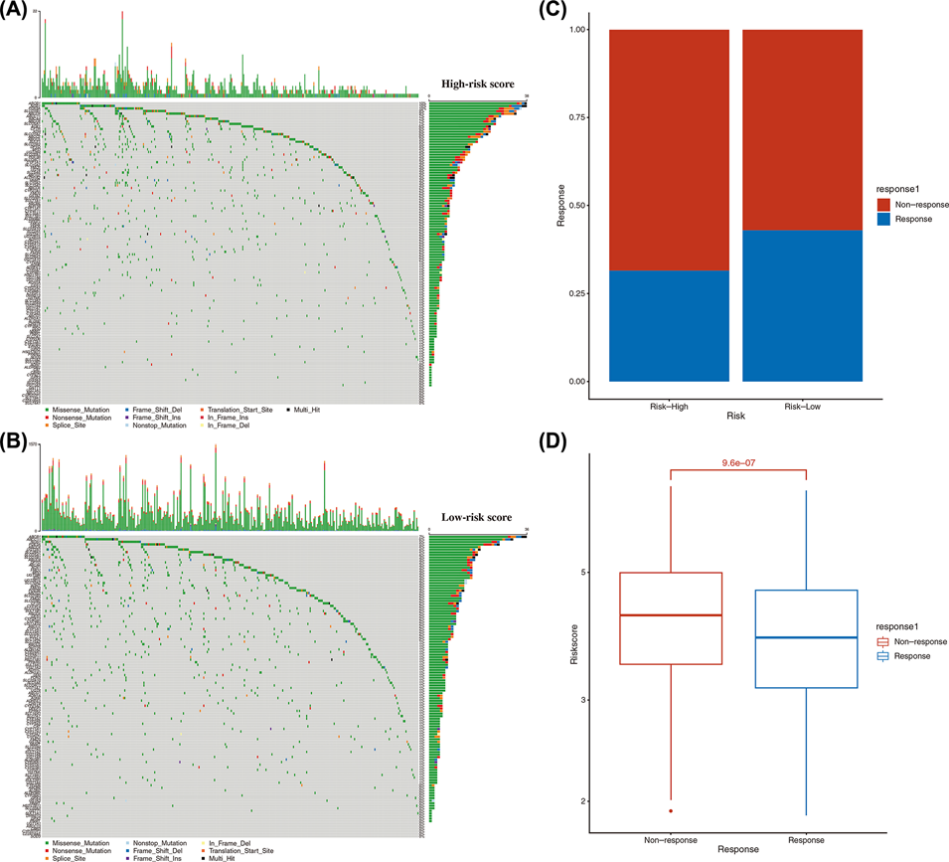
Mutation analysis and evaluation of medication reactions in the high-and low-risk score groups (**A**) Mutation frequency and mutation type of 118 candidate ADME genes in the high-risk score group (the intersection of the DEGs and ADME genes between tumor and normal tissues in the validation set). (**B**) Mutation frequency and mutation type of 118 candidate ADME genes in the low-risk score group. (**C**) Distribution of treatment outcomes in samples in the high- and the low-risk score groups. The *X*-axis represents the sample groups; the *Y*-axis is the proportion of samples in groups of treatment outcomes. (**D**) Distribution of risk score of samples with different treatment outcomes. The *X*-axis represents the treatment outcome groups; the *Y*-axis is the risk score.

The mutation analysis of 118 candidate ADME genes (the intersection of the DEGs and ADME genes between tumor and normal tissues in the training set) in the high- and the low-risk score groups showed that such genes were at low mutation levels in both the high- and low-risk score groups. The mutation frequency of candidate ADME genes in the high-risk score group was lower than in the low-risk group (Figure 12A). The main mutation type in the low-risk score group was a missense mutation (Figure 12B).

The results of evaluations of medication reactions in the training set showed significant differences in immunotherapy between the high- and the low-risk score groups. The samples in the low-risk group predicted a higher proportion of treatment responses, which indicated that the low-risk group tended to have better treatment outcomes (Figure 12C). In addition, comparing the risk scores of the groups with different treatment outcomes showed significant differences in the risk scores between the groups with different curative effects, indicating that the samples with good treatment effects tended to be in the low-risk score group (Figure 12D).

## Discussion

Lung cancer is a common malignant tumor with a complicated pathogenesis, a high mortality rate, and a poor prognosis; it is a serious threat to global public health [9]. Although major breakthroughs have been achieved in the treatment of lung cancer-especially for patients with NSCLC who can receive new targeted and immune drugs-these new treatments have problems, such as limited benefits and drug resistance [3]. Additional studies on the interaction mechanism between drugs and tumors are urgently needed to clarify the influencing factors of drug metabolism, improve the early diagnosis of lung cancer, predict the drug response in patients, and predict the long-term survival of patients. Because of the gradual popularization of next-generation sequencing, more genes associated with drug metabolism have been discovered, and this discovery provides the possibility of extended study in related fields [10].

ADME genes represent a group of genes involved in the ADME of drugs [4,5,11,12]. Typical phase I (functionalization) enzymes are oxidases, dehydrogenases, hydrolases, and deaminases [13]. Phase II (conjugation) drug-metabolizing enzymes are mainly transferases, such as UDP-glucuronosyltransferases, sulfotransferases, and thiopurine methyltransferase [14]. Drug transporters include the solute carrier transporters (SLC15A2, SLC22A2, etc.) and the ATP-binding cassette transporters (ABCB1, ABCG2, etc.); they are involved in the drug uptake into cells [15]. ADME genes can affect the metabolism of many endogenous and exogenous substances, which can affect the genesis and progression of cancer through DNA damage, control of the growth signaling pathway, and other direct actions. Like the key roles of ADME genes in metabolism, elimination of anticancer drugs, and cancer regulation, many genetic polymorphisms of ADME genes, such as SNPs, are closely related to carcinogenesis and drug reactions [16–18]. Recently, one study has shown significant differences in the expression of ADME genes between lung cancer tumor tissues and normal tissues [8]; some ADME genes have been significantly correlated with the prognosis of patients with lung cancer [8].

In the present study, we obtained the DEGs between NSCLC tumor tissues and normal tissues with publicly available database information. After obtaining the intersection between DEGs and ADME genes, univariate Cox and lasso regression analyses were performed. Seven prognosis-associated ADME genes were identified to establish a risk prediction model. Among these genes, *SLC16A1* plays a tumor suppressor role in NSCLC, liver cancer, and oral squamous cell carcinoma [19–21]. *SLC7A5* is an anti-glutamine transporter and is important in models of colorectal tumorigenesis in early and late metastatic diseases [22]. One Study have shown that *SLCO1B1* variation can be used as a biomarker of breast cancer response to simvastatin [23]. The gene polymorphisms of *CYP24A1* and *CYP17A1* have been closely correlated with the risk of liver, lung, stomach, and prostate cancers in a Chinese population [24,25]. Another study has shown that the expression of *ABCC8* mRNA can be used as an independent prognostic index in patients with glioma and can predict the sensitivity of gliomas to temozolomide [26].

Using the risk score formula, a risk score value was calculated for each patient in the training set, and these values provided a median value for the model. According to the median risk value, NSCLC samples were divided into a high-risk group and a low-risk group. The Kaplan–Meier curve, the ROC curve, the validation set data, and other data were used to evaluate the efficiency of the risk prediction model. Univariate and multivariate Cox regression analyses were performed on the model's risk score and clinical features. The risk score was an independent prognostic factor of OS in NSCLC.

We carried out an in-depth study on the high- and the low-risk score groups with GO and KEGG analyses, which found that the DEGs in the two groups were enriched in metabolism-related molecular pathways and tumor-related pathways. ssGSEA analysis and quantification of an immune cell infiltration score and immune-related functions revealed a high infiltration level of M2 macrophages in the high-risk score group in the training set, suggesting the possibility of tumor immunosuppression. The results of TIDE analysis showed that the effect of immunotherapy in the high-risk group may be poor. The results of mutation difference analysis showed no significant difference between the high- and low-risk groups. The influence of 118 candidate ADME genes on the risk value of the samples was not highly correlated with gene mutation. Therefore, we can we have shown that the gene mutation has no regulatory effect on the risk value, so our findings which exclude a direction of gene mutations as a topic for continued analysis.

## Conclusion

In conclusion, the prognostic model established with the seven ADME genes in the present study has good predictive ability for the prognosis of patients with NSCLC. The model’s risk score is an independent prognostic factor for OS in patients with NSCLC. This model reflects the immune status of patients with NSCLC well and evaluates drug reactions according to the high- and the low-risk score groups. However, the present study also has some limitations. For example, our prediction model is based on retrospective data from a public database, so large-scale prospective studies are needed to verify its clinical utility. In addition, these bioinformatics findings must be verified by experimental research.

## Supplementary Material

Supplementary Materials S1-S3

## Data Availability

The datasets used and/or analyzed during the present study are available from the corresponding author upon reasonable request.

## Abbreviations

ADME: absorption, distribution, metabolism, and excretion
AUC: area under the curve
BP: biological processes
CC: cellular component
DEG: differentially expressed gene
GEO: Gene Expression Omnibus
GO: Gene Ontology
KEGG: Kyoto Encyclopedia of Genes and Genomes
LASSO: least absolute shrinkage and selection operator
MF: molecular function
NK: natural killer
NSCLC: non-small-cell lung cancer
OS: overall survival
PCA: principal component analysis
PPI: protein–protein interaction
ROC: receiver operating characteristic
ssGSEA: single-sample gene set enrichment analysis
t-SNE: t-distributed stochastic neighbor embedding
TCGA: The Cancer Genome Atlas
TNM: tumor node metastasis
