# Supplementary material for: Establishment of non-small-cell lung cancer risk prediction model based on prognosis-associated ADME genes
Source: Biosci Rep. 2021 Oct 14;41(10):BSR20211433. doi: 10.1042/BSR20211433 (PMC8527211; doi:10.1042/BSR20211433)
Supplement: Supplementary Materials S1-S3 [file BSR-2021-1433_supp.zip › BSR-2021-1433_suppS1.docx]

**supplementary material 1. Clinical features of patients with NSCLC in TCGA cohort**

| Clinical information | No. of patients |
| --- | --- |
| **Total** | 1019 |
| **Gender** |  |
| male | 610（59.9%） |
| female | 409（40.1%） |
| **Age** |  |
| ≤60 | 268（26.3%） |
| ＞60 | 751（73.7%） |
| **Pathologic-T** |  |
| T1T2 | 859（84.3%） |
| T3T4 | 160（15.7%） |
| **Pathologic-N** |  |
| N0N1 | 898（88.1%） |
| N2N3 | 121（11.9%） |
| **Pathologic-M** |  |
| MX | 219（21.5%） |
| M0 | 768（75.4%） |
| M1 | 32（3.1%） |
| **Pathologic-stage** |  |
| Stage I/II | 983（96.5%） |
| Stage III/IV | 36（3.5%） |
